# Book Notices

**Published:** 1895-11

**Authors:** 


### Book Notices.

INTERNATIONAL CLINICS.—A Quarterly of Clinical Lectures on Medicine, Neurology, Surgery, Genito-Urinary Surgery, Gynecology, Obstetrics, Ophthalmology, Laryngology, Pharyngology, Rhinology, Otology, and Dermatology, by Professors and Lecturers in the leading Medical Colleges of the United States, Germany, France, Great Britain and Canada. Edited by Judson Daland, M. D. (Univ. of Penn.), Philadelphia, Instructor in Clinical Medicine and Lecturer on Physical Diagnosis in the University of Pennsylvania, Etc., Etc. J. Mitchell Bruce, M. D., F. R. C. P., London, England, Physician to and Lecturer on the Principles and Practice of Medicine in the Charing Cross Hospital. David W. Finlay, M. D., F. R. C. P., Aberdeen, Scotland, Professor of Practice of Medicine in the University of Aberdeen; Physician to, and Lecturer on Clinical Medicine in, the Aberdeen Royal Infirmary, Etc. Price per Volume, Cloth, $2.75; Half Morocco, $3.00. J. B. Lippincott Company, Publishers, Philadelphia. Volume II, Fifth Series, 1895.

This is a most excellent collection of clinical lectures delivered at the various hospitals in this country and Europe by such men as Drs. A. Baginsky, E. H. Bradford, D. R. Brower, J. M. Baldy, A. Morgan Cartledge, Henry C. Coe, John B. Hamilton, H. A. Hare, J. W. Hulke, George Klemperer, I. N. Love, E. E. Montgomery, Edmund Owen, William L. Rodman, Robert Saundby, G. E. De Schweinitz, Wharton Sinkler, C. L. Schleich, E. L. Tompkins, William H. Wathen, Hal C. Wyman, and twenty-one other prominent physicians and surgeons.

To the regular readers of the "Clinics" it is unnecessary to say that these lectures are not dry dissertations on medical and surgical subjects, but every one is a practical clinic, the patients being introduced, their clinical history elicited, a diagnosis made in each case, and proper treatment, medical or surgical, given. If the case be a surgical one, each step of the operation is faithfully described—not what should be, but what is done—until the operation is completed and the wound is properly dressed.

In the "Clinics" we get the latest and best treatment in disease, and the most approved surgical methods and technique, the best men in the profession being our teachers. The "Clinics" is always good, but this volume, we think, surpasses any that has preceded it. H.

